# The relationship between demographic features, anthropometric parameters, sleep duration, and physical activity with ECG parameters in Fasa Persian cohort study

**DOI:** 10.1186/s12872-021-02394-8

**Published:** 2021-12-07

**Authors:** Alireza Mirahmadizadeh, Mojtaba Farjam, Mehdi Sharafi, Hossein Fatemian, Maryam Kazemi, Kiarash Roustai Geraylow, Azizallah Dehghan, Zahra Amiri, Sima Afrashteh

**Affiliations:** 1 Non-Communicable Diseases Research Center, Shiraz University of Medical Sciences, Shiraz, Iran; 2 Noncommunicable Diseases Research Center, Fasa University of Medical Sciences, Fasa, Iran; 3 Student Research Committee, Shiraz University of Medical Sciences, Shiraz, Iran; 4 Student Research Committee, Semnan University of Medical Sciences, Semnan, Iran; 5 Noncommunicable Diseases Research Center, Hormozgan University of Medical Sciences, Hormozgan, Iran; 6 Department of Public Health, School of Public Health, Bushehr University of Medical Sciences, Bushehr, Iran

**Keywords:** Anthropometric parameters, Sleep duration, ECG parameters, Cohort

## Abstract

**Backgrounds:**

Cardiovascular Diseases (CVDs) are the first leading cause of death worldwide. The present study aimed to investigate the relationship between demographics, anthropometrics, sleep duration, physical activity, and ECG parameters in the Fasa Persian cohort study.

**Methods:**

In this cross-sectional study, the basic information of 10,000 participants aged 35–70 years in the Fasa cohort study was used. The data used in this study included demographic data, main Electrocardiogram (ECG) parameters, anthropometric data, sleep duration, and physical activity. Data analysis was performed using t-test, chi-square, and linear regression model.

**Results:**

Based on multivariate linear regression analysis results, increased age was significantly associated with all study parameters. Nevertheless, gender and body mass index showed no significant relationship with SV3 and PR. Wrist circumference, hip circumference and waist circumference significantly increased the mean values of the ECG parameters. However, sleep duration was not significantly associated with the ECG parameters. In addition, hypertension was major comorbidity, which was shown to increase the mean values of the ECG parameters.

**Conclusion:**

Several factors affected the ECG parameters. Thus, to interpret ECGs, in addition to age and gender, anthropometric indices, physical activity, and previous history of comorbidities, such as hypertension and ischemic heart disease, should be taken into consideration.

## Introduction

Cardiovascular Diseases (CVDs) are one of the first leading causes of death worldwide [1]. In 2016, it was estimated that 17.9 million people died from CVDs, accounting for 31% of all global deaths [2]. In addition to high mortality, CVDs have considerable complications and are a major cause of certain disabilities, especially in old ages [3]. Therefore, the establishment of screening programs among high-risk populations aimed at reducing the mortality rate is of paramount importance [4]. Electrocardiography (ECG), also known as an electrocardiogram, is an affordable, non-invasive, easy-to-apply, and widely used method to screen heart diseases [5]. Experimental evidence has shown that many CVDs can be effectively diagnosed, controlled, and prevented through continuous monitoring and analysis of ECG signals [6]. Therefore, monitoring physiological signals, such as ECG signals, is a comprehensive technique for evaluating, controlling, and preventing CVDs [7]. However, the role of ECG in CVDs screening is still controversial and under debate in many studies [8].

Several authors have defined population-based reference values based on specific populations. However, it is obvious that ECG screening is influenced by various factors, including demographics and anthropometrics [9–11]. Previous studies have shown that males had prolonged PR intervals, wider QRS duration, and shorter QTC intervals than females. In addition, older age was associated with longer PR intervals and wider QRS duration in both genders. At the same time, higher Body Mass Index (BMI) was linked to prolonged PR interval, wider QRS duration, and significantly negative QRS axis^0^ [12]. Generally, identifying and managing patients with coronary artery disease is highly dependent on ST wave changes, T-wave inversion, and Q-wave presentation. Less than 50% of people with Myocardial Infarction (MI) showed ST-wave changes in their initial ECG [13]. T-wave changes were also shown to be associated with myocardial ischemia, and T-wave inversion was an important indication of this disease [14].

Furthermore, ECG abnormalities were associated with an increased risk of adverse cardiac events, including hypertrophic cardiomyopathy [15], tachycardia, prolonged PR interval, QT inversion, and abnormal changes in ECG axes [16]. Moreover, elevated PR interval increased atrial fibrillation and MI risk in the elderly population [17]. Furthermore, the results of the previous studies demonstrated that higher BMI was positively associated with QRS duration, indicating that higher weight increased the odds of ventricular depolarization disturbances [12]. Dhingra and colleagues also disclosed that increased QRS duration was positively associated with left ventricular hypertrophy, with a significantly stronger association amongst obese males [18].

ECG screening is commonly used to identify pathological processes, such as lethal arrhythmias associated with short- or long-term QT intervals. Although such conditions rarely occur in young and healthy populations, early detection can prevent the catastrophic complications of sudden cardiac death [8]. However, there is limited information on the relationship between demographic features, anthropometric parameters, sleep duration, physical activity, and ECG parameters on the middle-aged population; therefore, our study in Iran to fill this gap evaluates the middle-aged population of the Fasa Persian cohort.

## Methods

In this cross-sectional study, basic information of the participants enrolled in the Fasa cohort study was used. This cohort consisted of 10,000 people aged 35–70 years and was conducted in the Shashdeh region with 24 villages. The study data, including demographics, socioeconomic status, routine tests, sample preparation in biobanks, physical examinations, anthropometric measurements, physical activity, sleep duration, nutritional status, and ECG, were stored as online digital information for each individual [19]. The required data included ECG parameters such as QT interval duration (ms), QRS duration (ms), QRS axis^0^, PR interval (ms), RaVL amplitude (mv), and SV3 amplitude (mv), demographics including age, gender, education level, and marital status, anthropometrics like BMI, wrist circumference, hip circumference, and waist circumference, physical activity [Metabolic Equivalent of Task (MET)], and the 24-h sleep time, which were selected from the basic cohort information. Out of the 10,000 participants of the cohort, only 7119 had recorded ECGs. All these participants had 12-lead ECGs before the interview. They were asked to shave the precordium area for better attachment of the ECG electrodes. A standard twelve-lead electrocardiogram taken from all participants, who were asked to shave their pericardium before performing electrocardiography; an electrocardiogram was performed by an expert technician and saved in the health level 7 standard format. ECG_S_ was initially interpreted by the application software (Cardiax®, version 3.50.2, International Medical Equipment Developing Co. Ltd., Budapest, Hungary) and transferred to the central data collection software. Then the initial interpretation includes hear rate, intervals, durations, analysis of vector, st-t change and voltage amplitude Approved by a cardiologist [19].

The IPAQ questionnaire was used to measure physical activity, and the Pittsburgh Questionnaire (PSQI) was used to assess participants' sleep. To measure weight, a scale was used to measure height and height, waist circumference, wrist circumference, and hip circumference. It should be noted that the scales used and meters were calibrated daily. Also, all measurements were performed by one person in one day.

This study is in agreement with the Helsinki declaration and Iranian national guidelines for ethics in research. (Reference number: IR.FUMS.RES.1394.3), and informed written consent was obtained from all participants.

The quantitative results were expressed as mean and standard deviation and the qualitative results as frequency and percentage. Independent t-test and chi-square tests were used to compare the variables by gender. Additionally, the one-dimensional linear regression model was used, and the variables with a significance level of less than 0.25 were entered into the multivariate model to eliminate the effect of confounders. Due to the presence of collinearity between the anthropometric variables and physical activity in the multivariate model, other variables were controlled and were separately entered into the model. The significance level was set at 0.05. The Kolmogorov test was used to evaluate normality. To conduct statistical analysis, STATA software version 12 was used.

## Results

A total of 7119 participants were recruited into this study, 3092 ones of whom (43.43%) were male. The mean age of the participants was 48.59 ± 9.34 years, and there was no significant age difference between males and females. A comparison of the characteristics of all study participants by gender has been presented in Table 1. The results revealed significant differences between males and females for all variables, except for age and sleep duration (*p* < 0.001).

**Table 1:** The demographic characteristics and the correlated variables by gender

Characteristics	Total population (n = 7119)		*P*-value	N
	Male	Female		7119
	n (%) 3092 (4343)	n (%) 4027 (5657)		
Age	48.46 ± 9.28	48.69 ± 9.39	0.3052	48.59 ± 9.34
BMI	24.11 ± 4.44	26.84 ± 4.90	< 0.001	25.66 ± 4.89
Years of education	5.78 ± 4.04	3.73 ± 3.37	< 0.001	4.62 ± 3.81
*Marital status*				
Single	61 (1.97)	196 (4.86)	< 0.001	257 (3.61)
Married	3020 (97.67)	3263 (81.02)		6283 (88.26)
Widowed or divorced	11 (0.35)	568 (14.10)		579 (8.13)
Wrist circumference	17.26 ± 1.24	16.26 ± 1.26	< 0.001	16.69 ± 1.34
Hip circumference	97.24 ± 7.59	101.04 ± 9.27	< 0.001	99.39 ± 8.79
Waist circumference	89.32 ± 10.96	96.36 ± 11.52	< 0.001	93.30 ± 11.81
MET	2675.20 ± 831.52	2307.99 ± 408.48	< 0.001	2467.48 ± 654.03
*Diabetes*				
No	2839 (91.81)	3379 (83.90)	< 0.001	6218 (87.37)
Yes	251 (8.11)	648 (16.09)		899 (12.63)
*Hypertension*				
No	2733 (88.38)	2934 (72.85)	< 0.001	5667 (79.63)
Yes	357 (11.54)	1093 27.14)		1450 (20.37)
*Cardiac ischemic disease*				
No	2816 (91.07)	3498(86.86)	< 0.001	6314 (88.72)
Yes	274 (8.86)	529 (13.13)		803 (11.28)
Sleep duration	7.67 ± 1.85	7.71 ± 1.89	0.391	7.69 ± 1.87
Hr	66.00 ± 10.91	75.17 ± 11.60	< 0.001	71.19 ± 12.18
PR interval (ms)	140.55 ± 36.63	136.94 ± 32.31	< 0.001	138.51 ± 34.29
QRS duration (ms)	98.51 ± 10.69	95.47 ± 9.90	< 0.001	96.79 ± 10.36
QTc interval (ms)	420.28 ± 31.85	439.67 ± 33.70	< 0.001	431.25 ± 34.28
QRS axis^0^	38.53 ± 43.25	32.38 ± 31.76	< 0.001	35.05 ± 37.31
RaVL amplitude (mv)	0.249 ± 0.229	0.323 ± 0.228	< 0.001	0.291 ± 0.231
SV3 amplitude (mv)	− 0.128 ± 0.201	− 0.125 ± 0.201	< 0.001	− 0.126 ± 0.201

*BMI* body mass index, *MET* metabolic equivalent of task

### Correlation between age and ECG parameters

The results of the linear regression model showed that age was positively associated with ECG parameters, including PR (ms) (B = 0.275, *p* < 0.001), QRS (ms) (B = 0.047, *p* = 0.005), QTC (B = 0.331, *p* < 0.001), and RaVL (mm) (B = 0.003, *p* < 0.001). As such, increase in age by one year after the age of 35 years increased the average ECG parameters, including PR (0.275), QRS (0.047), QTC (0.331), and RaVL (0.003 m/s), but significantly decreased SV3 (mm) (b = − 0.002, *p* < 0.001) and QRS axis (b = − 0.454, *p* < 0.001) (Tables 2, 3, 4, 5, 6 and 7).

**Table 2:** Linear regression analysis of the association between PR interval (ms) and independent variables

Variables	Univariate			Multivariate		
	B	95% CI	*P*-value	B	95% CI	*P*-value
Age	0.298	0.213, 0.383	< 0.001	0.275	0.203, 0.347	< 0.001
*Gender*						
Male	Ref					
Female	− 3.61	− 5.21, − 2.00	< 0.001	− 6.63	− 7.85, − 5.42	< 0.001
*Marital status*						
Unmarried	Ref					
Married	3.11	− 1.159, 7.398	0.153	− 0.200	− 2.98, 2.580	0.888
Widowed or divorced	3.40	− 1.63, 8.44	0.185	− 0.083	− 3.40, 3.23	0.961
BMI	0.261	0.098, 0.423	0.002			
Education level	− 0.307	− 0.515, − 0.098	0.004	0.016	− 0.153, 0.185	0.851
Wrist circumference	1.75	1.169, 2.348	< 0.001	1.50	0.860, 2.14	< 0.001
Hip circumference	0 .149	0.058, 0.239	0.001	0.253	0.158, 0.348	< 0.001
Waist circumference	0.133	0.066, 0.201	< 0.001	0.190	0.143, 0.236	< 0.001
MET	0.047	− 0.025, 0.121	0.202	0.018	− 0.031, 0.069	0.471
*Diabetes*						
No	Ref					
Yes	2.69	0.298, 5.09	0.028	.087	− 1.50, 1.68	0.914
*Hypertension*						
No	Ref					
Yes	3.691	1.714, 5.668	< 0.001	2.40	0.963, 3.83	0.001
*Cardiac ischemic disease*						
No	Ref					
Yes	2.08	− 0.430, 4.60	0.104	0.687	− 1.01, 2.39	0.429
Sleep duration	− 0.097	− 0.521, 0.326	0.651	–	–	–

*BMI* body mass index, *MET* metabolic equivalent of task

**Table 3:** Linear regression analysis of the association between QRS duration (ms) interval and independent variables

Variables	Univariate			Multivariate		
	B	95% CI	*P*-value	B	95% CI	*P*-value
Age	0.103	0.078, 0.129	< 0.001	0.047	0.014, 0.080	0.005
*Gender*						
Male	Ref					
Female	− 3.03	− 3.51, − 2.55	< 0.001	− 4.070	− 4.63, − 3.50	< 0.001
*Marital status*						
Unmarried	Ref					
Married	3.395	2.10, 4.68	< 0.001	1.18	− 0.107, 2.468	0.072
Widowed or divorced	2.578	1.058, 4.098	0.001	1.099	− 0.437, 2.635	0.161
BMI	0.148	0.099, 0.197	< 0.001	0.235	0.184, 0.286	< 0.001
Education level	− 0.063	− 0.126, -.0003	0.049	− 0.066	− 0.145, 0.011	0.095
Wrist circumference	1.20	1.02, 1.38	< 0.001	0.855	0.665, 1.046	< 0.001
Hip circumference	0.101	0.073, 0.128	< 0.001	0.151	0.123, 0.179	< 0.001
Waist circumference	0.070	0.050, 0.090	< 0.001	0.101	0.080,0 .123	< 0.001
MET	0.021	− 0.0005, 0.043	0.055	− 0.0006	− 0.024, 0.022	0.958
*Diabetes*						
No	Ref					
Yes	0.696	− 0.028, 1.42	0.060	0.053	− 0.685, 0.792	0.887
*Hypertension*						
No	Ref					
Yes	1.762	1.165, 2.358	< 0.001	0.998	0.334, 1.66	0.003
*Cardiac ischemic disease*						
No	Ref					
Yes	2.994	2.23, 3.75	< 0.001	2.21	1.42, 3.007	0.429
Sleep duration	− 0.084	− 0.212, 0.044	0.198	0.025	− 0.103, 0.155	0.693

*BMI* body mass index, *MET* metabolic equivalent of task

**Table 4:** Linear regression analysis of the association between QTC interval (ms) and independent variables

Variables	Univariate			Multivariate		
	B	95% CI	*P*-value	B	95% CI	*P*-value
Age	0 .412	0.328, 0.497	< 0.001	0.331	0.225, 0.437	< 0.001
*Gender*						
Male	Ref					
Female	19.38	17.84, 20.93	< 0.001	16.82	15.03, 18.62	< 0.001
*Marital status*						
Unmarried	Ref					
Married	− 3.25	− 7.52, 1.009	0.135	− 1.82	− 5.97, 2.31	0.387
Widowed or divorced	6.24	1.21, 11.26	0.015	− 3.98	− 8.92, 0.967	0.115
BMI	0.899	0.738, 1.061	< 0.001	0.316	0.150, 0.482	< 0.001
Education level	− 1.17	− 1.37, − 0.963	< 0.001	− 0.093	− 0.345, 0.159	0.470
Wrist circumference	− 1.35	− 1.94, − 0.760	< 0.001	1.32	0.712, 1.940	< 0.001
Hip circumference	0.369	0.279, 0.460	< 0.001	0.152	0.061, 0.243	0.001
Waist circumference	0.455	0.388, 0.521	< 0.001	0.168	0.099, 0.238	< 0.001
MET	− 0.490	− 0.563, − 0.418	< 0.001	− 0.234	− 0.308, − 0.159	< 0.001
*Diabetes*						
No	Ref					
Yes	7.40	5.01, 9.80	< 0.001	0.251	− 2.14, 2.64	0.837
*Hypertension*						
No	Ref					
Yes	10.97	9.01, 12.93	< 0.001	2.423	0.280, 4.56	0.027
*Cardiac ischemic disease*						
No	Ref					
Yes	8.08	5.56, 10.59	< 0.001	1.961	− 0.587, 4.51	0.131
*Sleep duration*	− 0.012	− 0.437, 0.412	0.955	–	–	–

*BMI* body mass index, *MET* metabolic equivalent of task

**Table 5:** Linear regression analysis of the association between QRS axis^0^ and independent variables

Variables	Univariate			Multivariate		
	B	95% CI	*P*-value	B	95% CI	*P*-value
Age	− 0.560	− 0.651, − 0.468	< 0.001	− 0.454	− 0.572, − 0.336	< 0.001
*Gender*						
Male	Ref					
Female	− 6.15	− 7.89, − 4.40	< 0.001	− 8.43	− 10.55, − 6.31	< 0.001
*Marital status*						
Unmarried	Ref					
Married	− 3.10	− 7.75, 1.55	0.191	2.618	− 2.00, 7.23	0.267
Widowed or divorced	− 6.96	− 12.44, − 1.48	0.013	5.00	− 0.50, 10.51	0.075
BMI	− 1.51	− 1.68, − 1.34	< 0.001	− 1.42	− 1.61, − 1.24	< 0.001
Education level	0.876	0.649, 1.10	< 0.001	0.003	− 0.277, 0.285	0.979
Wrist circumference	− 2.27	− 2.91, − 1.62	< 0.001	− 3.45	− 4.14, − 2.76	< 0.001
Hip circumference	− 0.601	− 0.699, − 0.503	< 0.001	− 0.598	− 0.700, − 0.496	< 0.001
Waist circumference	− 0.637	− 0.709, − 0.565	< 0.001	− 0.553	− 0.630, − 0.475	< 0.001
MET	0.223	0.143, 0.303	< 0.001	0.096	0.013, 0.179	0.023
*Diabetes*						
No	Ref					
Yes	− 7.27	− 9.87, − 4.66	< 0.001	− 0.058	− 2.72, 2.60	0.966
*Hypertension*						
No	Ref					
Yes	− 11.83	− 13.96, − 9.69	< 0.001	− 3.84	− 6.23, − 1.45	0.002
*Cardiac ischemic disease*						
No	Ref					
Yes	− 8.45	− 11.18, − 5.72	< 0.001	− 1.49	− 4.33, 1.33	0.301
*Sleep duration*	0.090	− 0.372, 0.552	0.702	–		

*BMI* body mass index, *MET* metabolic equivalent of task

**Table 6:** Linear regression analysis of the association between RaVL amplitude (mv) and independent variables

Variables	Univariate			Multivariate		
	B	95% CI	*P*-value	B	95% CI	*P*-value
Age	0.004	0.003, 0.004	< 0.001	0.003	0.002, 0.004	< 0.001
*Gender*						
Male	Ref					
Female	0.073	0.062, 0.083	< 0.001	0.096	0.083, 0.108	< 0.001
*Marital status*						
Unmarried	Ref					
Married	0.037	0.008, 0.066	0.011	− 0.017	− 0.044, 0.009	0.206
Widowed or divorced	0.093	0.059, 0.127	< 0.001	− 0.017	− 0.049, 0.014	0.294
BMI	0.018	0.017, 0.019	< 0.001	.016	.015, .017	< 0.001
Education level	− 0.007	− 0.009, − 0.006	< 0.001	− 0.0006	− 0.002, 0.000	0.424
Wrist circumference	0.027	0.023, 0.031	< 0.001	0.040	0.036, 0.044	< 0.001
Hip circumference	0.007	0.006, 0.007	< 0.001	0.006	0.006, 0.007	< 0.001
Waist circumference	0.007	0.007, 0.0079	< 0.001	0.006	0.006, 0.007	< 0.001
MET	− 0.002	− 0.002, − 0.001	< 0.001	− 0.0005	− 0.001, − 0.00003	0.038
*Diabetes*						
No	Ref					
Yes	0.072	0.056, 0.088	< 0.001	− 0.002	− 0.018, 0.012	0.731
*Hypertension*						
No	Ref					
Yes	0.137	0.124, 0.150	< 0.001	0.081	0.067, 0.095	< 0.001
*Cardiac ischemic disease*						
No	Ref					
Yes	0.086	0.069, 0.103	< 0.001	0.022	0.005, 0.039	0.011
Sleep duration	− 0.001	− 0.004, 0.001	0.202	0.001	− 0.001, 0.003	0.465

*BMI* body mass index, *MET* metabolic equivalent of task

**Table 7:** Linear regression analysis of the association between SV3 amplitude (mv) and independent variables

Variables	Univariate			Multivariate		
	B	95% CI	*P*-value	B	95% CI	*P*-value
Age	− 0.002	− 0.003, − 0.002	< 0.001	− 0.002	− 0.002, − 0.001	< 0.001
*Gender*						
Male	Ref					
Female	0.003	− 0.006, 0.012	0.505	–	–	–
*Marital status*						
Unmarried	Ref					
Married	− 0.018	− 0.043, 0.006	0.147	0.005	− 0.019, 0.030	0.666
Widowed or divorced	− 0.022	− 0.052, 0.007	0.137	0.025	− 0.004, 0.055	0.100
BMI	− 0.003	− 0.004, − 0.002	< 0.001	− 0.003	− 0.004, − 0.002	< 0.001
Education level	0.003	0.002, 0.004	< 0.001	− 0.0001	− 0.001, 0.001	0.876
Wrist circumference	− 0.008	− 0.012, − 0.005	< 0.001	− 0.008	− 0.012, − 0.005	< 0.001
Hip circumference	− 0.001	− 0.001, − 0.0005	< 0.001	− 0.001	− 0.001, − 0.0006	< 0.001
Waist circumference	− 0.001	− 0.001, − 0.001	< 0.001	− 0.001	− 0.001, − 0.0007	< 0.001
MET	0.0002	− 0.0002, 0.0006	0.307	–	–	–
*Diabetes*						
No	Ref					
Yes	− 0.019	− 0.033, − 0.005	0.006	0.008	− 0.006, 0.023	0.255
*Hypertension*						
No	Ref					
Yes	− 0.054	− 0.066, − 0.043	< 0.001	− 0.030	− 0.043, − 0.017	< 0.001
*Cardiac ischemic disease*						
No	Ref					
Yes	− 0.040	− 0.054, − 0.025	< 0.001	− 0.010	− 0.025, 0.005	0.189
Sleep duration	0.002	− 0.0003, 0.004	0.090	0.0002	− 0.002, 0.002	0.854

*BMI* body mass index, *MET* metabolic equivalent of task

### The relationship between gender and ECG parameters

The results of multivariate analysis showed that gender was significantly associated with all ECG parameters, except for S in V3. After adjusting for the effects of other variables, the average intervals between PR, QRS, and QRS axis parameters were reduced by − 6.03, − 4.070, and − 8.43 units, respectively, among females (*p* < 0.001). Females also displayed an increased QTC interval and RaVL amplitude by 16.82 and 0.096 units, respectively, compared to males (*p* < 0.001) (Tables 2, 3, 4, 5, 6 and 7).

### The relationship between marital status and ECG parameters

According to the results presented in Tables 2, 3, 4, 5, 6 and 7, marital status showed no significant association with any of the ECG parameters (*p* > 0.05) (Tables 2, 3, 4, 5, 6 and 7).

### The relationship between BMI and ECG parameters

After controlling the effects of other variables, BMI was significantly associated with R in Avl, S in V3, QRS axis, QTC, and QRS. Accordingly, an increase in BMI by one point elevated the average interval of R in aVL (0.016, *p* < 0.05), QTC (0.316, − 1.42), and QRS (0.235, *p* < 0.001), but decreased the interval of S in V3(− 0.003) and QRS axis (− 1.42) (*p* < 0.001) (Tables 2, 3, 4, 5, 6 and 7).

### The relationship between education level and ECG parameters

The effect of education level in the multivariate model showed that this variable was not significantly linked to any of the ECG parameters (*p* > 0.05) (Tables 2, 3, 4, 5, 6 and 7).

### The relationship between wrist circumference and ECG parameters

The present study's findings showed a significant relationship between wrist circumference and PR, QRS, S in V3, QRS axis, QTC, and R in aVL. Based on the results, a one cm increase in wrist circumference increased the average interval of PR, QRS, QTC, and R in aVL by 1.501, 0.855, 1.32, and 0.040 units, respectively (*p* < 0.001). An increase in wrist circumference by one cm also resulted in a decrease in the QRS axis and S in V3 by 3.45 and 0.008 units, respectively (*p* < 0.001) (Tables 2, 3, 4, 5, 6 and 7).

### The relationship between the hip circumference and ECG parameters

The results indicated that hip circumference was significantly associated with S in V3, R in aVL, QRS, QTC, and PR parameters. After controlling the effects of other variables, an increase in hip circumference by one centimeter increased the mean interval of R in aVL, QTC, QRS, and PR parameters by 0.006, 0.152, 0.151, and 0.253, respectively (*p* < 0.001), but decreased the mean interval of S in V3 and QRS axis by − 0.001 and − 598, respectively (*p* < 0.001).

### The relationship between waist circumference and ECG parameters

Among Iranian adults, waist circumference was shown to be significantly associated with all ECG parameters. After controlling the effects of other variables, an increase in waist circumference by one centimeter increased the mean interval of PR (0.190), QRS (0.101), and R in aVL (0.006) (*p* < 0.001) but decreased the mean interval of QTC (− 0.234), QRS axis (− 0.553), and S in V3 (− 0.001) (*p* < 0.001) (Tables 2, 3, 4, 5, 6 and 7).

### The relationship between MET and ECG parameters

In the present study, MET showed a significant relationship with QTC, QRS, and R in aVL. Accordingly, an increase in MET by one unit decreased the mean interval of QTC and R in aVL by − 0.234 and − 0.0005, respectively (*p* < 0.05), but increased the mean interval of QRS axis by 0.096 (*p* = 0.023) (Tables 2, 3, 4, 5, 6 and 7).

### The relationship between diabetes and ECG parameters

Among the participants of this cohort, diabetes was not significantly associated with any of the ECG parameters in the multivariate model (*p* > 0.05) (Tables 2, 3, 4, 5, 6 and 7).

### The relationship between hypertension and ECG parameters

In this study, after adjusting for the effects of other variables, hypertension showed a significant relationship with PR, QRS, S in V3, QRS axis, QTC, and R in aVL. Accordingly, PR, QRS, QTC, and R in aVL increased by 2.40, 0.998, 2.423, and 0.081 units, respectively, among hypertensive individuals (*p* < 0.05). Besides, the mean intervals of QRS and S in V3 parameters were reduced by 3.48 and − 0.030 units, respectively, in hypertensive people compared to non-hypertensive ones (*p* < 0.05) (Tables 2, 3, 4, 5, 6 and 7).

### The relationship between cardiac ischemic disease and ECG parameters

The patients with cardiac ischemic disease showed a significant relationship only with R in aVL. The mean interval of R in aVL increased by 0.022 in these patients compared to the healthy individuals after controlling the effects of other variables (*p* = 0.011). This variable showed no significant associations with other ECG parameters (*p* > 0.05) (Tables 2, 3, 4, 5, 6 and 7).

### The correlation between the total amount of sleep duration in 24 h and ECG parameters

Among the Iranian adults of this cohort, the sleep duration was not significantly associated with any of the ECG parameters in the multivariate model (*p* > 0.05) (Tables 2, 3, 4, 5, 6 and 7).

## Discussion

This cross-sectional study was conducted on 7119 individuals aged 35–75 years who participated in the Fasa cohort study. This study is among the few studies describing each individual ECG parameter's associations with demographic features, anthropometric parameters, sleep hours, and physical activity. The present study results suggested that an increase in age increased the QRS, PR, QT, and R in aVL intervals. Numerous studies on ECG changes in different age groups have also shown that the PR, QRS [8], and QT [20]. Intervals were shortened by age. Since the risk of MI, atrial fibrillation, and left ventricular hypertrophy increases with age and prolongation of PR and QRS interval duration may manifest in the presence of these diseases [12, 18], the present study results were expected and justifiable. In the current research, QRS, PR, and QRS axis intervals were shorter in females than in males, which was consistent with the results of other studies [11, 12, 21]. Hence, physicians should pay attention to gender and gender differences in ECG interpretations. A cohort based on 3777 old-aged Asians found longer PR interval, wider QRS, shorter QTc interval, and taller SV3 in men. Moreover, age increase was associated with longer PR interval, wider QRS, larger R aVL, and more leftward QRS axis [12]. The P wave in ECG reflects the atrial activation, which occurs first in the right and then in the left atrium, creating a positive upward wave in leads I, II, AVL, and AVF. Atrial repolarization creates a small wave that is not detectable on ECG because it is covered by a QRS wave. The QRS wave represents ventricular activation, and the ventricular repolarization is highlighted by the T wave. Changes in the amplitude or interval of the waves, even in a normal range can indicate mechanical or electrical pathology of the heart [22].

The results also demonstrated that QRS, QT, and R in aVL intervals increased with elevated BMI. These results agreed with those of other studies regarding the relationship between obesity and QRS, QT, and PR prolongation [9, 23]. Similar results were reported by Maruyama et al. [24], who found that lengthening of PR interval and QRS duration and the leftward shift of the QRS axis was associated with BMI increment. Prolonged PR can be a sign of myocardial hypertrophy, while it is asymptomatic in many cases and occurs due to anatomical changes of the chest in obese people [23]. The present study's findings also showed that the elevation of other anthropometric indices, such as wrist circumference, waist circumference, and hip circumference, was associated with increased QRS, PR, and R in V1 in ECG. Waist circumference, one of the widely used criteria for metabolic syndrome, is one of the most common determinants of ECG changes and hypertension. Abdominal obesity raises the diaphragm, resulting in increased sympathetic activity and cardiac output, thereby affecting the ECG parameters [25, 26]. An Asian cohort showed that the participants with higher BMIs had a longer PR interval, wider QRS, a more negative QRS axis, and a larger RaVL [12]. A cross-sectional study on 11,308 participants also indicated an increase in BMI and central obesity increased PR interval and P wave duration [27]). Braschi et al. [28]. found that the QT interval and QTc interval significantly increased in obese compared to normal-weight participants. However, they revealed no association between obesity and QT dispersion and QT apex dispersion.

Similarly, Fraley et al. [10] reported prolonged QT interval duration and QTc interval, leftward shifts of the P wave, QRS, and T wave axes, and various markers of left ventricular hypertrophy among the obese participants. Another study conducted on rats demonstrated that there were no rhythm disturbances early in obesity. The results only revealed an increase in the resting heart rate. The observed ECG abnormalities suggest ventricular changes related to impaired myocardial depolarization, repolarization, and cardiac morphology changes [29].

Exercise and physical activity has been known to be protective factor against CVDs. However, athletes and individuals who have constant and intense physical activity, it causes structural and electrical changes in the heart, including bradycardia sinus, first-degree Atrioventricular (AV) block, and early repolarization displayed in the heart the heart ECG. Other less common changes in athletes' ECGs include prolongation or shortening of QT, pathological Q-wave, changes in the ST segment, and enlargement of the right atrium [30].

The results from the Atherosclerosis Risk in Communities (ARIC) Study showed that Obesity and extreme weight change to be risk factors for incident AF, and being physically active to be a protective factor for AF [31]. In addition, obesity and hypertension were shown to impact P wave indices such as prolonging the PR interval, P wave maximum duration, and P wave terminal force, which was not supported by our findings [32].

In the current investigation, participants with higher physical activity, evaluated by a higher MET score, had a higher QRS axis, shorter QTc interval, and shorter R wave amplitude in aVL. Nevertheless, no significant association was reported between sleep duration and the ECG parameters. It has been expressed that regular physical activity positively affects the vagal activity on the heart [33]. Lawan et al. [34] reported a higher prevalence of sinus bradycardia, first-degree AV block, and right and left axis deviation in athletes compared to non-athletes. They also found the right bundle branch block, prolonged QT interval, and elevation of the J point only among the athletes. On the contrary, Melanson EL et al. [35] stated that heart rate and Heart Rate Variability (HRV) were highly reproducible regardless of the physical activity level. Their results suggested that although HRV might be greater in physically active participants than in inactive individuals, no dose-dependent association was found between the physical activity level and HRV.

The present study results showed a longer PR interval, QTc interval, and QRS complex, higher R wave amplitude in aVL, lower QRS axis, and S wave amplitude in V3 among the hypertensive participants compared to the non-hypertensive ones. Ch. Sia et al. [8] disclosed that systolic blood pressure > 140 mmHg or diastolic blood pressure > 90 mmHg independently increased the prevalence of increased QRS voltage on ECG. This can be explained by the effect of high blood pressure on the heart's structure, causing left ventricular hypertrophy over time, which presents as QT prolongation in the ECG [28]. In contrast to our findings, results from the Multi-Ethnic Study of Atherosclerosis (MESA) suggest that higher systolic BP, diastolic BP, and pulse pressure were associated with greater P wave terminal force, not with PR interval or P wave duration [36].

In general, patients with diabetes are at a higher risk for ischemic heart disease due to macro and microvascular complications, which can be completely asymptomatic in the normal 12-lead ECG due to diabetic neuropathy. However, nonspecific changes are usually observed in the ECGs of these patients. Tachycardia and shortening of QT and QRS intervals are examples of these changes that are justified by the increased sympathetic tone in these patients [37]. In the present study, diabetes was not associated with any significant changes in the ECG parameters. Noori et al. [37]. found higher QT interval, QTd interval, QTc interval, and QTcd interval in patients with diabetes. Most of the ECG abnormalities in patients with diabetes reported by the previous studies were more pronounced in patients with cardiac autonomic neuropathy.

In comparison to similar ECG changes in other diseases, ECG changes in patients with diabetes were not specific and were mainly caused by an increased tone of the sympathetic nervous system, which was indirectly confirmed by the heart rate variability findings in patients with diabetes [38]. In addition, hypertension and diabetes, as risk factors for CVDs, might cause major pathological changes in the ECG parameters by causing myocardial ischemia. The current study findings also indicated higher R wave amplitude in patients with ischemic heart disease.

Among the strengths of this study are the large sample size and study population. In addition, the effects of confounding factors, such as age, gender, physical activity, and comorbidities, were considered in the evaluation of changes in the ECG parameters. However, the study limitations included its cross-sectional design and recruitment of people aged over 35 years. Hence, the results are required to be confirmed in further longitudinal studies with longer follow-up periods on populations with a wider age range.

## Conclusion

In conclusion, the study findings revealed a correlation between the ECG parameters and anthropometric indicators, physical activity status, and previous history of such comorbidities as hypertension and ischemic heart disease. Hence, these factors have to be taken into consideration in the interpretation of ECG signals. Moreover, it is necessary to account for the factors associated with ECG parameters according to each population's racial and ethnic characteristics and provide normal and abnormal ECG interpretation implications as guidelines to physicians.

## Data Availability

The datasets generated and/or analyzed during the current study are not publicly available due to its being the intellectual property of Fasa University of Medical Sciences but are available from the corresponding author on reasonable request.
